# Accuracy of hydrocortisone dose administration via nasogastric tube

**DOI:** 10.1111/cen.13876

**Published:** 2018-11-09

**Authors:** Eleni Daniel, Martin J. Whitaker, Brian Keevil, Jerry Wales, Richard J. Ross

**Affiliations:** ^1^ The University of Sheffield Sheffield UK; ^2^ Manchester Academic Health Science Centre (MAHSC), The University of Manchester Manchester UK

**Keywords:** bioavailability, cortisol, hydrocortisone, nasogastric tubes, paediatric

## Abstract

**Objective:**

Hydrocortisone via nasogastric (NG) tube is used in sick children with adrenal insufficiency; however, there is no licensed formulation for NG administration.

**Methods:**

We investigated hydrocortisone recovery after passage through NG tubes in vitro for three formulations: liquid suspension, crushed tablets mixed with water, and hydrocortisone granules designed for oral administration to children. Cortisol was measured by LC‐MS/MS.

**Results:**

Hydrocortisone content was variable and recovery low after preparation in syringe and prior to passage through NG tubes. For doses, 0.5 and 2.0 mg mean percentage recovery was as follows: liquid suspension 57% and 58%; crushed tablets 46% and 30%; and hydrocortisone granules 78% and 71%. Flushing the administering syringe increased recovery. Hydrocortisone recovery after passage with flushing through 6‐12Fr gauge NG tubes was variable: liquid suspension 61%‐92%, crushed tablets 40%‐174%, hydrocortisone granules 61%‐92%. Administration of hydrocortisone granules occluded 6 and 8Fr NG tubes; however, administration using a sampling needle to prevent granules being administered gave a recovery of 74%‐98%.

**Conclusions:**

The administration of hydrocortisone through NG tubes is possible; however, current methods deliver a variable dose of hydrocortisone, generally less than that prescribed. Attention should be placed on the technique used to optimize drug delivery such as flushing of the administering syringe. Hydrocortisone granules block small NG tubes but behaved as well as the commonly used liquid suspension when prepared with a filtering needle that filters out granules.

## 1 INTRODUCTION

Long‐term treatment with hydrocortisone is required in children with adrenal insufficiency and treatment starts from birth in neonates with congenital adrenal hyperplasia. Replacement therapy with oral hydrocortisone is generally given in 3‐4 daily doses.^1^, ^2^, ^3^ The administration of oral hydrocortisone in young children may require a nasogastric (NG) tube during inter‐current illness, and treatment with hydrocortisone to reduce bronchopulmonary dysplasia in premature infants is becoming more popular^4^; however, there are no licensed formulations for administration via NG tube.

Hydrocortisone is poorly soluble in aqueous solutions and the suspension is viscous and therefore its delivery may be adversely affected when intervening equipment such as syringes and NG tubes are used.^5^ Inaccuracy in the hydrocortisone dose delivered leads to under‐ or over‐ replacement and is associated with poor disease control and potentially poor long‐term health outcomes.^6^, ^7^, ^8^ For hydrocortisone administration via the NG route, the preparations most commonly used in paediatric practice are liquid suspensions (syrup) available as special unlicensed formulations and tablets crushed into a fine powder and mixed with water.^9^, ^10^, ^11^, ^12^ A multi‐particulate immediate‐release formulation of hydrocortisone has been specifically developed for oral administration to neonates, infants and young children.^13^, ^14^ This study investigated the in vitro recovery of hydrocortisone after passage through NG tubes of varying bore for three different hydrocortisone preparations; a liquid suspension (Rosemont Pharmaceuticals Ltd, Braunton, UK), crushed hydrocortisone tablets mixed with water (Auden McKenzie (Pharma Division) Ltd, Barnstaple, UK) and hydrocortisone granules (Alkindi, Diurnal Ltd, Cardiff, UK).

## 2 METHODS

### 2.1 ***Protocol development and hydrocortisone formulations tested***

The experimental protocol was developed following consultation with adult and paediatric endocrine specialist nurses, senior neonatal intensive care nurses, paediatric pharmacists and a review of current clinical practice.^9^, ^15^, ^16^, ^17^, ^18^ In children, oral hydrocortisone is usually given in 3‐4 daily doses from 0.5 mg upwards with the commonest dose being 2 mg,^2^, ^3^, ^13^, ^19^ so we chose to test doses of 0.5 and 2.0 mg (=target doses). Current practice in our institution is to use either liquid in suspension (100 mL bottle at 5 mg/5 mL) or crushed 10 mg hydrocortisone tablets. When using NG tubes in neonates, the total drug administration volumes should be minimal with NG flushes up to 2 mL,^15^, ^17^ so we chose to give doses in maximum 2 mL volume with 2 mL flush in the NG tubes 6‐8Fr that are used in this age group. The protocol was tested on the bench multiple times, timed and supervised by a paediatric endocrine nurse to ensure compliance with clinical practice. Two researchers performed the experiments and all stages were timed for standardization.

### 2.2 Protocol for preparation of hydrocortisone formulations for administration

- Liquid hydrocortisone suspension: the bottle (100 mL bottle at 5 mg/5 mL) was shaken vigorously and the required amount drawn into a sterile 10 mL syringe.
- Hydrocortisone tablets: one 10 mg tablet was crushed using a tablet crusher into a fine powder, 10 mL of sterile water were added and mixed and the required amount drawn into a sterile 10 mL syringe.
- Hydrocortisone granules: the contents of one capsule (0.5 mg or 2 mg) were suspended in 2 mL sterile water in a 10 mL sterile syringe, the suspension was shaken vigorously for 30 seconds, left on the bench for 15 minutes and then shaken again for 30 seconds.

### 2.3 Hydrocortisone recovery at the nasal end of the NG tube

The experiment assessed the recovery of hydrocortisone in samples prepared for NG administration (but not administered) according to the above protocol. There were two parts in this experiment. In the first part two target doses, 0.5 and 2 mg were prepared as above and then expelled into bijou tubes. Six repeats were performed. The second part assessed whether the suboptimal recovery of hydrocortisone was due to dose remnants in the administering syringe: a second set of samples for the liquid suspension formulation was collected that included flushing of the administering syringe with 2 mL water. The flushing liquid was collected together with the sample for hydrocortisone quantification and three repeats were performed. The samples were stored at 4°C prior to analysis.

### 2.4 Hydrocortisone administration through NG tubes

Nasogastric tubes come in variable sizes and are measured using the French (Fr) scale, with smaller French values representing a narrower diameter and shorter length. A size 6Fr NG is used for long‐term feeding in a small neonate and 12Fr is the adolescent and young adult size.^16^ Medicines and fluids are administered at the nasal end of the NG tube and exit through a small ovoid opening next to the gastric end. The administration of all three preparations was tested using transparent 6, 8, 10 and 12Fr NG tubes to cover the size range used across the paediatric population. Each NG tube was held in a ring stand, at a height of 30 cm, with the lower end in a collecting tube. Each formulation was administered from the 10 mL syringe used for preparation and using the same syringe each tube was then flushed with water (2 mL for the 6Fr and 8Fr tubes, 5 mL for the 10Fr tubes and 10 mL for the 12Fr tubes). The NG tubes were left to drain all administered materials into the collecting tube at the gastric end of each NG for 3 minutes (NG‐passage sample). The experiment was repeated six times. Following hydrocortisone granules administration only, the NG tubes were observed for the presence of granules intraluminally. If any granules were present, the tube was flushed once more 30 minutes later; the patency of the tube was recorded but the liquid was not added to the previously collected NG‐passage sample.

### 2.5 Alternative method for preparation of hydrocortisone granules for administration

A second method of sample preparation for hydrocortisone granules was developed to test the feasibility of neonatal size (6Fr) NG administration and assess the recovery of hydrocortisone. In a bijou tube, 2 mg of hydrocortisone granules were suspended in 2 mL sterile water, the suspension was shaken vigorously for 30 seconds and allowed to rest on the bench for 0, 15, 30, 45 or 60 minutes, then shaken vigorously again for 30 seconds. Immediately afterwards, 1 mL of suspension (target dose 1 mg) was aspirated into a 2.5 mL sterile syringe through a metallic sampling needle used for aspirating drugs for oral administration that prevented any granules from entering the syringe (Nutrisafe 2 sampling needle external diameter 1.1 and 52 mm long, Vygon Ltd, UK). The needle was then removed and the contents of the syringe were either pushed down a 6Fr NG tube or expelled into a small bijou tube (control sample). With the same syringe, 2 mL sterile water was aspirated and then flushed into the NG tube (NG passage sample) or expelled into the control samples. The NG tubes were left to drain into the bijou tubes for 3 minutes and the experiment was repeated five times.

### 2.6 Quantification of hydrocortisone by liquid chromatography tandem mass spectrometry

All samples were labelled with a numerical code, stored at 4°C and transferred on ice for liquid chromatography‐tandem mass spectrometry (LC‐MS/MS) analysis of hydrocortisone at the Biochemistry Department, Manchester University NHSA Foundation Trust. Prior to analysis the samples were warmed in a hot bath for 5 minutes, shaken and a 1:10 000 dilution with water was made. The LC‐MS/MS method has been described elsewhere^20^ but briefly, standard, quality control or hydrocortisone sample (20 μL) was manually pipetted directly into the well of a 96‐deep well block (Thermo, Hemel Hempstead, UK). To this, 40 μL of 0.1 mol/L zinc sulphate was added. This was vortexed for 10 seconds followed by the addition of 100 μL of internal standard. The block was heat‐sealed (Thermo) and vortexed for 1 minutes, then centrifuged at 8000 *g* for 5 minutes. Following centrifugation, the plate was transferred directly to the autosampler for analysis; 10 μL of sample was injected into the liquid chromatography (LC) system using partial loop mode. LC‐MS/MS was performed using an Acquity I Class coupled to a XEVO TQ‐D detector (Waters, Wilmslow, UK). The quantity of hydrocortisone in mg in each sample was calculated from the hydrocortisone concentration. The inter‐assay imprecision (%CV) was 13%, 9% and 5% at concentrations of 100, 400 and 800 nmol/L, respectively. The intra‐assay imprecision was 12%, 7% and 9%.

### 2.7 Data presentation and statistical analysis

Results are shown as mean ± SD of the repeats. The data are expressed either as mean hydrocortisone content in mg or % hydrocortisone recovery, that is, the mean hydrocortisone content in each set expressed as a percentage of the dose administered (target dose). ANOVA with multiple comparisons was used for the analysis of differences between the three hydrocortisone formulations and between the bench time rest periods allowed for the alternative preparation method for hydrocortisone granules suspension (GraphPad 7, GraphPad Software, La Jolla CA). Unpaired two‐tailed *t* tests were performed for comparison of pre‐ and post‐NG administration recovery for each time point in the alternative method of preparation. A *P* value of <0.05 was considered significant.

## 3 RESULTS

### 3.1 Recovery of hydrocortisone prior to NG administration

**Figure 1:**
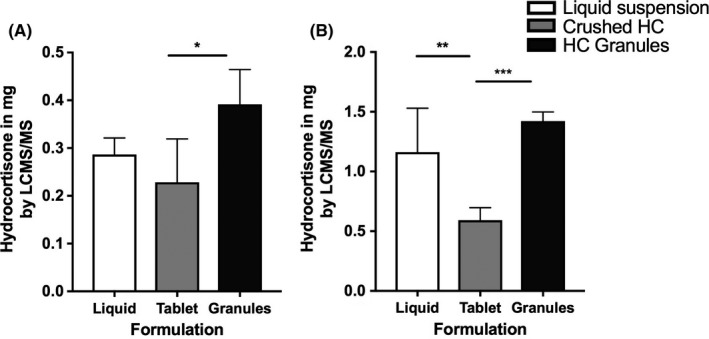
Mean hydrocortisone content prior to NG tube administration. Three hydrocortisone formulations (liquid suspension, crushed 10 mg tablets, and hydrocortisone granules) were prepared in syringes at 0.5 & 2.0 mg absolute dose and then expelled into a universal tube with hydrocortisone content in universal measured by LC‐MS/MS (A) 0.5 mg dose (B) 2.0 mg dose (^*^ *P* = 0.004, ^**^ *P* = 0.001, ^***^ *P* < 0.001)

The recovery of hydrocortisone from all three preparations at the nasal end of the NG tube prior to NG administration was low: mean ± SD % recovery of target dose for doses 0.5 and 2.0 mg was; liquid suspension 57% ± 7% & 58% ± 18%, crushed hydrocortisone mixed with water 46% ± 18% & 30% ± 5%, hydrocortisone granules 78% ± 15% & 71% ± 4% (Figure 1). The delivery of hydrocortisone with hydrocortisone granules was significantly better than crushed hydrocortisone for the 0.5 mg dose (*P* < 0.01) and the 2 mg dose (*P* < 0.01), and the liquid suspension was better than the crushed hydrocortisone for the 2 mg dose (*P* < 0.01) (Figure 1).

**Figure 2:**
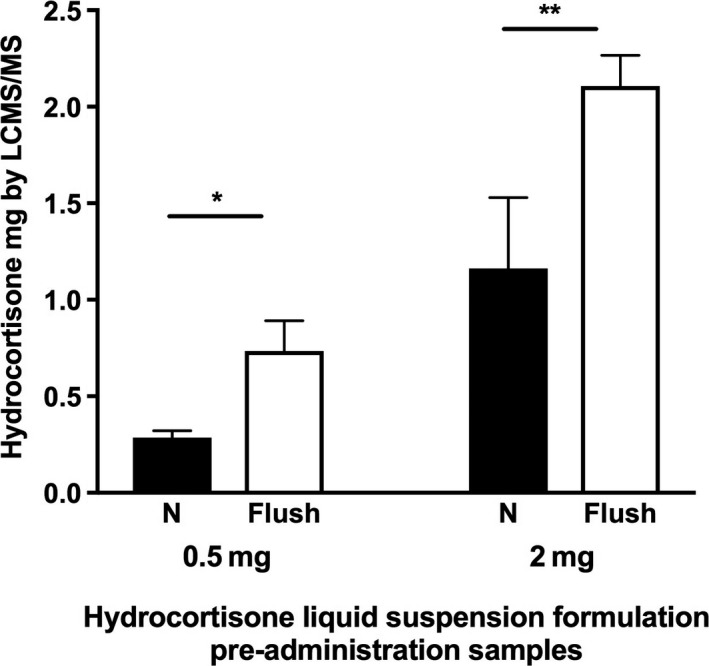
Mean hydrocortisone content prior to NG tube administration after flushing of the syringe used to draw up the dose (Flush: pre‐administration samples with 2 mL flushing of the administrating syringe, N, pre‐administration samples without flushing of the syringe, ^*^ *P* < 0.001, ^**^ *P* = 0.002)

The delivery of hydrocortisone in the pre‐administration samples of the liquid suspension increased significantly following flushing of the administrating syringe; mean ± SD % recovery of target dose; 0.5 mg dose without flushing 57% ± 7% vs with flushing 147% ± 31% (*P* < 0.01); 2 mg dose without flushing 58% ± 18% vs with flushing 105% ± 8% (*P* < 0.01, Figure 2). Based on these results syringes for all formulations were flushed for the experiments using the NG tubes.

### 3.2 Hydrocortisone recovery after passage through NG tubes

**Figure 3:**
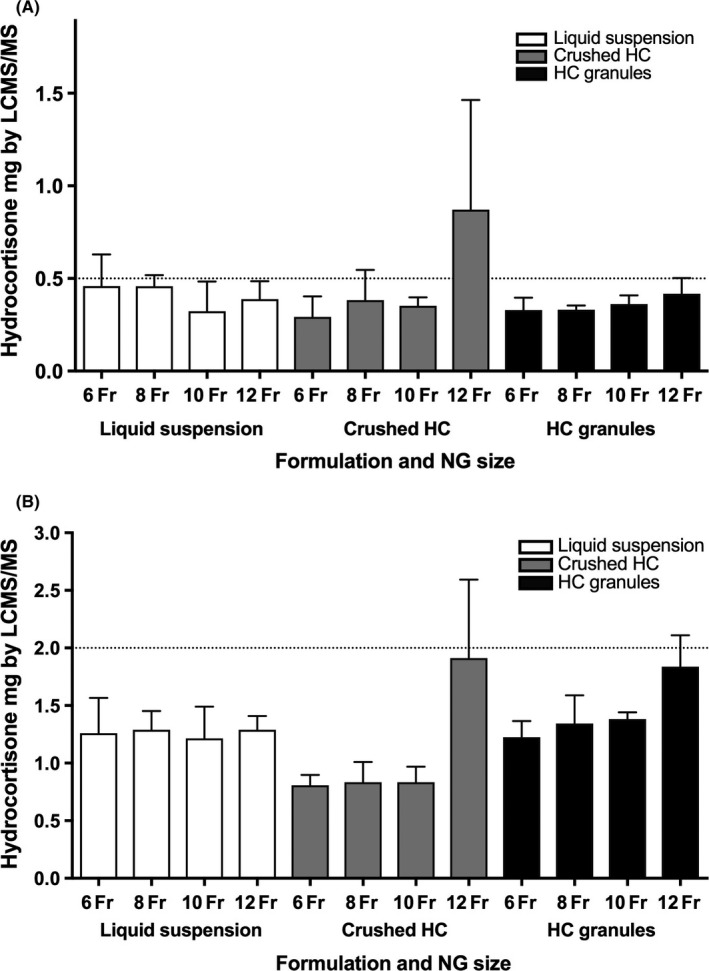
Mean hydrocortisone content after preparation in a syringe, administration through NG tubes gauge 6‐12Fr followed by flushing (A) Hydrocortisone dose 0.5 mg and (B) Hydrocortisone dose 2.0 mg

In this in vitro setting, it was possible to administer hydrocortisone through neonatal, paediatric and adolescent size NG tubes using all three preparations, although the delivery was variable. The hydrocortisone granules and the liquid suspension showed similar results throughout the range of NG tube sizes whereas the crushed hydrocortisone tablets gave greater variability for both doses (Figure 3, Table 1). The delivery of hydrocortisone for the 0.5 mg dose mean ± SD % recovery of target dose for the four different size NG tubes was; liquid suspension 65% ± 32% to 92% ± 34%, crushed hydrocortisone 59% ± 22% to 174% ± 118%, hydrocortisone granules 66% ± 13% to 83% ± 17% and for the 2 mg dose; liquid suspension 61% ± 14% to 65% ± 6%, crushed hydrocortisone 40% ± 5% to 96% ± 34%, hydrocortisone granules 61% ± 7% to 92% ± 14%.

**Table 1:** The recovery of hydrocortisone from three different hydrocortisone formulations administered through NG tubes, collected with water flush and quantified by LC‐MS

	0.5 mg dose			2 mg dose		
	Mean in mg	Standard deviation	% delivery of target dose	Mean in mg	Standard deviation	% delivery of target dose
Liquid suspension						
6Fr	0.46	0.17	92%	1.26	0.31	63%
8Fr	0.46	0.06	91%	1.29	0.16	64%
10Fr	0.32	0.16	65%	1.22	0.27	61%
12Fr	0.39	0.10	78%	1.29	0.12	65%
Crushed tablets						
6Fr	0.29	0.11	59%	0.81	0.09	40%
8Fr	0.38	0.16	77%	0.83	0.18	42%
10Fr	0.35	0.05	70%	0.83	0.14	42%
12Fr	0.87	0.59	174%	1.91	0.68	96%
Hydrocortisone granules						
6Fr	0.33	0.07	66%	1.23	0.14	61%
8Fr	0.33	0.02	66%	1.35	0.24	67%
10Fr	0.36	0.05	72%	1.38	0.06	69%
12Fr	0.42	0.09	83%	1.84	0.27	92%

**Figure 4:**
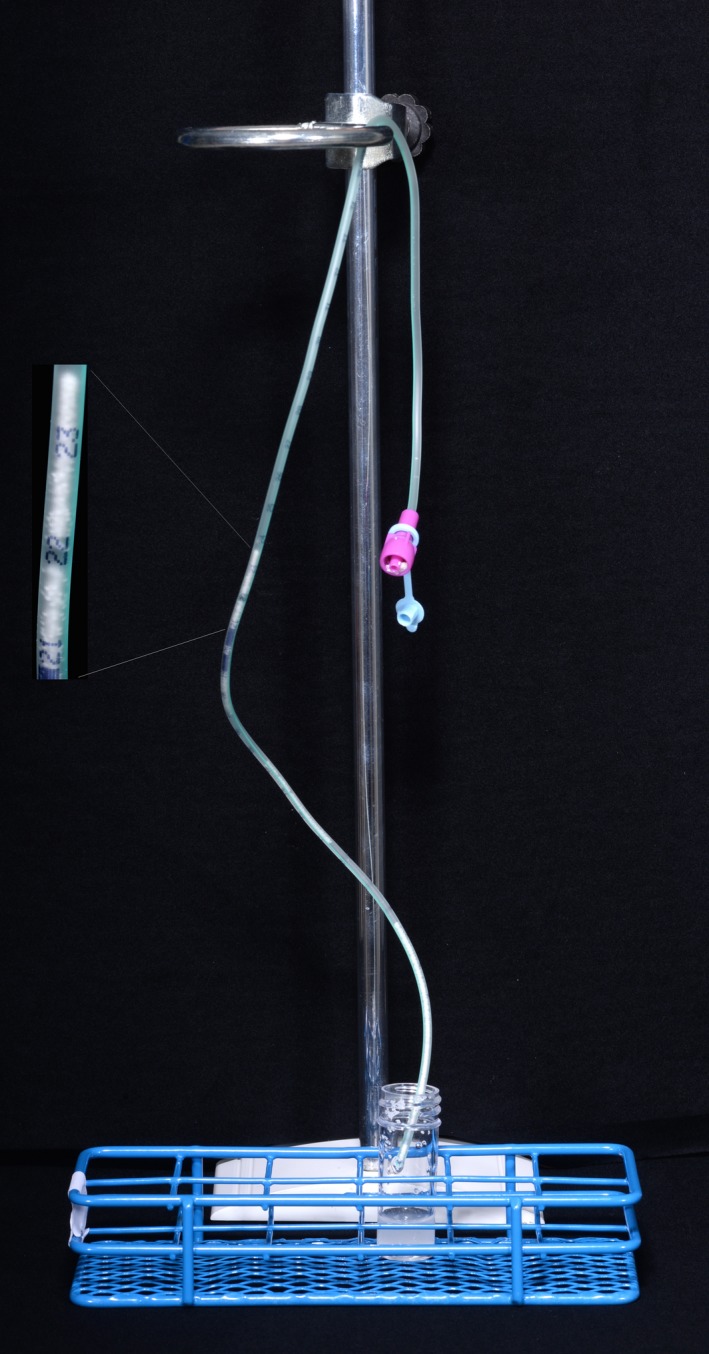
Hydrocortisone granules occluding 6Fr NG tube [Colour figure can be viewed at wileyonlinelibrary.com]

The possibility of mechanical tube occlusion due to administration of hydrocortisone granules was further explored. Following nasogastric administration of hydrocortisone granules, the NG tubes were observed for granules; no remaining granules were visible in the 10 and 12Fr. However, hydrocortisone granules were trapped within 6 and 8Fr tubes and the water flush did not remove them completely (Figure 4). Flushing the NG tubes immediately after administration of hydrocortisone granules was difficult although there was no complete occlusion of the NG tube during the administration phase. When NG tubes were left to drain for 30 minutes and a second flush was attempted complete occlusion was observed in 10% of 6Fr NG tubes and 50% of 8Fr NG tubes. Fewer granules were observed to enter the 6Fr NG tube compared to 8Fr tube.

### 3.3 Recovery of hydrocortisone from hydrocortisone granules using an alternative method of preparation

**Figure 5:**
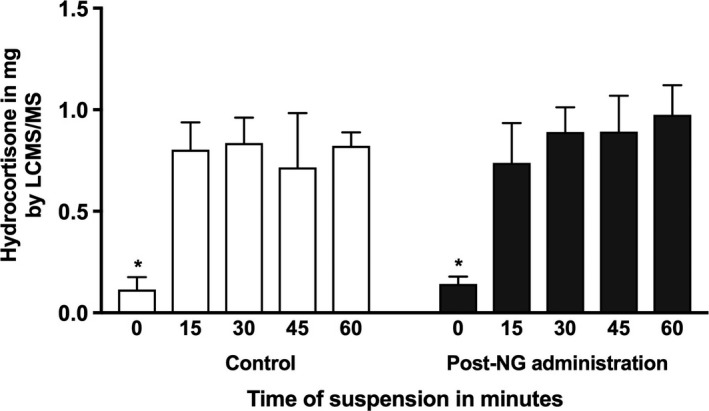
Recovery of hydrocortisone from hydrocortisone granule suspension in water (1 mg/mL) pre‐ and post‐administration through 6Fr gauge neonatal NG tube. Hydrocortisone granules were mixed with water and the samples were allowed 0, 15, 30, 45, and 60‐minute bench rest before aspiration of the required dose using a syringe connected to a sampling needle that excluded aspiration of granules (^*^ *P* < 0.001 ANOVA analysis, post‐hoc analysis shows significant difference between time 0 to all other time points)

To avoid granules entering the NG tube from the administering syringe, an alternative preparation method was developed and tested in the neonatal size (6Fr) NG tubes. Hydrocortisone granules were suspended in water for 0, 15, 30, 45 and 60 minutes to test if suspension time affected recovery. As shown in Figure 5, hydrocortisone recovery before and after administration down the NG tube was similar for each time point (*P* values 0.1 to 0.6). In the pre‐administration control set, hydrocortisone recovery between the different time points significantly increased between time zero and 15 minutes of bench suspension (*P* < 0.01) and the same was found for the post‐NG passage samples (*P* < 0.01, Figure 5). For post‐NG tube passage the recovery was as follows: 0 minutes 14% ± 4%, 15 minutes 74% ± 20%, 30 minutes 89% ± 12%, 45 minutes 89% ± 18%, 60 minutes 98% ± 15%. No NG tube blockages were observed with this method.

## 4 DISCUSSION

We have shown that it is possible to administer hydrocortisone via a nasogastric tube; however, dose recovery at the gastric end of the nasogastric tube is very variable and generally less than that administered. Three hydrocortisone formulations were tested: a liquid suspension (Rosemont Pharmaceuticals Ltd), crushed tablets mixed with water (Auden McKenzie (Pharma Division) Ltd) and hydrocortisone granules (Alkindi, Diurnal Ltd). At the nasal end of the NG tube, recovery was poor for all three formulations, between 30%‐78% unless the administering syringe was flushed. The recovery after passage down NG tubes with flushing was variable (40%‐174%) and generally <80% of the dose administered with the greatest variability seen for crushed tablets where in some cases recovery was <50% of the dose administered. Variability was least with hydrocortisone granules with recovery between 61% and 92%. Recovery of the dose administered was not affected by tube size for the liquid suspension but for crushed tablets and hydrocortisone granules recovery was best with the largest tube (12Fr). Hydrocortisone granules blocked the smaller NG tubes but this was avoided by generating a hydrocortisone suspension from the granules by leaving in water for 15 minutes and then using a sampling needle for drug aspiration that did not allow granules to be aspirated into the syringe.

Crushing oral medication to a fine powder is common practice for nasogastric administration in adults and children but it is an unlicensed use of the medication.^21^ Compounding from adult dose formulations is common in paediatrics when no dose appropriate formulation is available,^13^, ^22^ it is undertaken by pharmacy as well as carers and can lead to therapeutic failure among other risks.^23^ Capsules prepared by pharmacy from compounded hydrocortisone tablets have been found to have unacceptably variable drug content in over 20% of batches and have led to clinically and biochemically evident glucocorticoid overtreatment.^7^, ^22^ In our study, crushed hydrocortisone tablets mixed with water showed significant variability in the recovery of the administered hydrocortisone dose, usually with significantly low recovery but occasionally the recovery was above 100% of the target dose meaning that higher amount of hydrocortisone than the target dose (0.5 mg or 2 mg) was recovered in the sample. This likely reflects problems with the current practice of preparing small doses from 10 mg adult dose tablets. Another factor could be the loss of active pharmaceutical ingredient that could be up to 10% of the mass during hydrocortisone compounding because hydrocortisone sticks in the equipment used for compounding.^24^ Furthermore, hydrocortisone is relatively insoluble in water,^5^, ^25^ which means most hydrocortisone is in suspension not solution.

Few studies have reported the administration of medications through NG tubes and none have reported on hydrocortisone.^26^, ^27^, ^28^, ^29^ Our results show suboptimal recovery of hydrocortisone at the gastric end. High variability and low recovery of medications such as proton‐pump inhibitors administered through NG tubes were commonly observed in in vitro studies and recovery increased when higher volumes of solvent were used for drug dissolution prior to NG administration and flushing of the equipment.^17^, ^26^, ^27^, ^28^ Similar to our observation, formulations consisting of granules frequently cause NG tube obstructions.^30^, ^31^

It is important to follow appropriate techniques when administering medications down NG tubes and this applies to patients and carers who can be trained to give medications through NG tubes in the community. However, medicines are usually used out of license and there is lack of data on the accuracy of drug delivery through this method.^9^, ^16^, ^30^ We found that flushing the equipment (syringes) improves delivery for liquid suspension hydrocortisone. This has implications in children treated with hydrocortisone via the oral route when intervening equipment such as syringes are used; flushing of devices is important to maximize recovery for hydrocortisone, which is poorly soluble in water and sticks to plastics.^5^, ^24^ Our in vitro results demonstrate that specific methods need to be followed for different formulations of hydrocortisone to maximize recovery and accurate dosing and that most methods lead to under dosing.

Hydrocortisone granules have been recently licensed in Europe for replacement therapy of paediatric adrenal insufficiency and according to the summary of product characteristics they are not suitable for administration through nasogastric tubes.^13^ Consistent with this, we found that hydrocortisone granules blocked smaller NG tubes. Removing the granules by creating a suspension in a universal tube shaken and left for 15 minutes then aspirating using a sampling needle to avoid granules and administered down a NG tube resulted in a dose recovery of 74%‐98% which was comparable to and less variable than the other hydrocortisone formulations; however, this is not a licensed method of administration for hydrocortisone granules.

The strengths of this study are the protocol design that was developed to reflect current clinical practice in the administration of hydrocortisone in young children and the accurate method for estimating hydrocortisone concentration by liquid chromatography tandem mass spectrometry. The methods for the preparation of the three hydrocortisone formulations were different because we were comparing a liquid solution, tablets and granules that are available in clinical practice in different dose strengths (liquid 1 mg/mL vs tablets 10 mg vs granules 0.5 and 2 mg). These differences could affect the results, for example, the accuracy of hydrocortisone administration from crushed tablets might be better if a 5 mg tablet was used for the chosen target doses 0.5 and 2 mg rather than a 10 mg tablet; however, a 5 mg tablet is not available in Europe and therefore not tested. Two researchers performed the experiments and although the data were reviewed to check for operator‐dependent trends there was no formal statistical comparison between the two and this is a limitation of the study. This was an in vitro study and the concentration of hydrocortisone at the end of the NG tubes, however accurate, does not necessarily reflect the plasma concentrations in vivo and our results should be viewed in this light.

In conclusion, although delivery of hydrocortisone through NG tubes is possible, significant attention should be placed on the technique used to optimize drug delivery. The delivery of hydrocortisone with hydrocortisone granules was comparable with the currently used formulations and in fact granules seem to behave as well as the liquid suspension which is the current standard and most optimal formulation for oral administration; however, it leads to tube occlusions in the smaller gauge NG tubes (6 and 8Fr). Using a sampling needle to prevent the administration of granules is an alternative technique that delivers 74%‐98% of the required target dose.

## CONFLICT OF INTEREST

RJR & MJW are Directors of Diurnal Ltd. and hold stock. Diurnal Ltd. supplied Alkindi granules for this study. ED, BW and JW have nothing to disclose.
